# Rottlerin and Cancer: Novel Evidence and Mechanisms

**DOI:** 10.1100/2012/350826

**Published:** 2012-01-04

**Authors:** E. Maioli, C. Torricelli, G. Valacchi

**Affiliations:** ^1^Department of Physiology, University of Siena, Aldo Moro Street, 53100 Siena, Italy; ^2^Department of Biology and Evolution, University of Ferrara, Luigi Borsari Street 46, 44100 Ferrara, Italy; ^3^Department of Food and Nutrition, Kyung Hee University, Seoul 130-701, Republic of Korea

## Abstract

Because cancers are caused by deregulation of hundreds of genes, an ideal anticancer agent should target multiple gene products or signaling pathways simultaneously. Recently, extensive research has addressed the chemotherapeutic potential of plant-derived compounds. Among the ever-increasing list of naturally occurring anticancer agents, Rottlerin appears to have great potentiality for being used in chemotherapy because it affects several cell machineries involved in survival, apoptosis, autophagy, and invasion. The underlying mechanisms that have been described are diverse, and the final, cell-specific, Rottlerin outcome appears to result from a combination of signaling pathways at multiple levels. This paper seeks to summarize the multifocal signal modulatory properties of Rottlerin, which merit to be further exploited for successful prevention and treatment of cancer.

## 1. Introduction

Drug discovery from medicinal plants has played an important role in the treatment of cancer, and, indeed, a large majority of available anticancer drugs are natural products or natural product-derived drugs, or natural product mimics [1]. Emblematic examples of plant-derived compounds that entered in (phase I–III) clinical trials include curcumin, genistein, soy isoflavones, green tea/epigallocatechin gallate, and resveratrol [2]. These and other promising phytochemical agents, belonging to diverse structural and functional chemical classes, work by various mechanisms of action to prevent, arrest, or reverse either the initiation phase or the progression of carcinogenesis.

Because cancers are caused by deregulation of hundreds of genes [3], an ideal anticancer agent should target simultaneously multiple gene products or signaling pathways.

 Most of the plant-derived agents, which have been also studied by a mechanistic point of view, are signal transduction modulators, hormone modulators, anti-inflammatories, antimutagens, and antioxidants.

This review intends to present the multiple pharmacological properties of Rottlerin, an old/new natural substance that, over the years, has revealed a bewildering number of cellular and molecular targets, all potentially implicated in the control of (cancer) cell life and death.

## 2. The Source of Rottlerin

**Figure 1:**
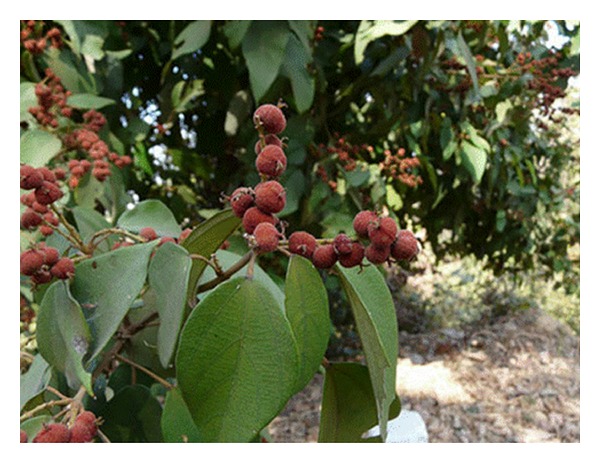
The Kamala tree.

The Mallotus Philippinensis, also known as Kamala Tree (Figure 1), grows in the tropical regions of India, Philippines, Southeast Asia, and Australia. This rain forest, evergreen tree produces a fruit that, when ripe (February and March), is covered with a red powder, which is collected by simply rolling the berries. This powder, called kamala, is used locally to produce an orange-brown die for coloring textiles and, suspended in water, mucilage or syrup, is also used as an old folk remedy against tape-worm, because of its laxative effect [4]. Though no scientific records exist, other folkloric uses include various afflictions of the skin, particularly scabies and herpetic ringworm, in which Kamala is used as a topical remedy. The powder is also used in treating eye diseases, bronchitis, abdominal disease, spleen enlargement and other illnesses, and legend says it is a powerful aphrodisiac [5].

 In more recent history, phloroglucinol derivatives extracted from various parts of the Kamala tree have been demonstrated to have antifertility actions [6] and antiallergic properties [7].

**Figure 2:**
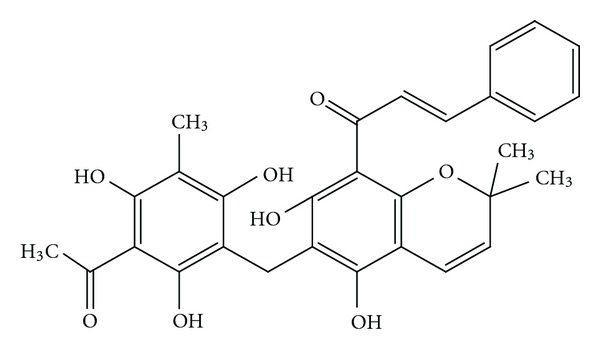
The Rottlerin structure.

Rottlerin, also called mallotoxin (Figure 2), is the principal phloroglucinol constituent of kamala and can be extracted, purified, and concentrated from the powder [8]. The IUPAC name for Rottlerin is (E)-1-[6-[(3-acetyl-2,4,6-trihydroxy-5-methylphenyl)methyl]-5,7-dihydroxy-2,2-dimethylchromen-8-yl]-3-phenylprop-2-en-1-one. The molecular formula for the structure is C_30_H_28_O_8_ and the structure has a molecular weight of 516.53852 g/mol.

 Commercially available Rottlerin has a purity of 85 to 99%, depending on the companies (Sigma, Calbiochem, Biomol, etc.). Rottlerin is supplied as an orange-brown powder, soluble in DMSO, chloroform, or ethanol, insoluble in water. Rottlerin is not an approved drug although it shows a low toxicity profile in an animal model of Parkinson (mice) [9]. In this study, both intraperitoneal (3–7 mg/Kg) and oral (20 mg/Kg) administration, exhibited protective effects and was not toxic. In addition, HPLC measurements revealed that the drug reached the target tissues in an intact and active form (1125 pg/mg brain tissue).

## 3. Rottlerin Usage: The Open Debate on Selectivity

The commercial production of Rottlerin, for in vitro uses only, began in 1994, following the paper by Gschwendt et al. reporting that PKC*δ* is selectively inhibited by 3–6 *μ*M Rottlerin 5–30-fold stronger than other PKCs at the same concentrations [10].

Since then, many studies of PKC*δ* signaling have used Rottlerin as a specific inhibitor and much of what is known regarding the involvement of PKC*δ* in a variety of biological processes derived from such studies.

However, most of these studies should be interpreted with skepticism because shortly thereafter, it was demonstrated that Rottlerin has no direct effect on PKC*δ* kinase activity in vitro [11]. In addition, Rottlerin was found to inhibit many other protein kinases, such as PRAK, MAPKAP-2, Akt/PKB, and CaMK [12].

Moreover, in an illuminating study, Soltoff demonstrated that Rottlerin uncouples mitochondrial respiration from oxidative phosphorylation thereby reducing ATP levels and affecting several cellular functions [13]. The author concluded that “the concept that Rottlerin is a specific inhibitor of PKC*δ* should be challenged and changed within the entire scientific community. It appears to be entirely without merit to continue to conclude reflexively that its mechanism of action is due to its direct inhibition of PKC*δ* activity.”

On the basis of these critical aspects about the application of Rottlerin as a PKC*δ* inhibitor, LC Laboratories discontinued selling Rottlerin.

Soltoff's study provided an instructive insight into the ability of Rottlerin to modulate several biological and biochemical processes in a PKC*δ*-independent way. For instance, in pancreatic acinar cells, 6 *μ*M Rottlerin (Calbiochem) depletes ATP levels, thus preventing the phosphorylation of many signaling proteins and inhibiting enzymatic secretion and several intracellular pathways, in a PKC*δ*-independent manner. Consistently, the studied inhibitory effects of Rottlerin, in pancreatic acini were mimicked by the mitochondrial uncouplers CCCP and FCCP [14].

Nonetheless, many studies of PKC*δ* signaling have used Rottlerin in conjunction with other methods, such as overexpression of constitutively activated PKC*δ* or downregulation by dominant-negative PKC*δ* and PKC*δ*-small interfering RNA, obtaining consistent and convincing results.

In order to resolve the question of whether Rottlerin is a PKC*δ* inhibitor, Soltoff suggested that, although Rottlerin is not effective in inhibiting PKC*δ* activity in vitro, the reduction of ATP levels can block PKC*δ* tyrosine phosphorylation and indirectly inhibit its translocation and kinase activity in cultured cells [15].

Alternatively, it is possible that Rottlerin, although not directly on PKC*δ*, can produce cellular changes that mimic those produced by the direct inhibition of PKC*δ*. In this regard, an emblematic example of convergent but independent effects of Rottlerin and PKC*δ* downregulation is the recent study by Matta et al. [16] performed in chicken chondrogenic mesenchymal cells. The authors found that both PKC*δ* silencing and 2.5–10 *μ*M Rottlerin (Sigma) decreased the protein levels of Sox9, the major cartilage-specific transcription factor, and impaired cartilage matrix production. However, they concluded that the inhibition of cartilage formation in the Rottlerin-treated cells is probably PKC*δ* independent for two main reasons: (i) they failed to unambiguously demonstrate inhibition of PKC*δ* activity by Rottlerin and (ii) Rottlerin decreased while PKC*δ* silencing increased the phosphorylation status of ERK1/2, one of the key regulators that influence in vitro chondrogenesis.

It is quite surprising that, despite the warning about the lack of specificity and activity towards PKC*δ*, Rottlerin is still used as a selective PKC*δ* inhibitor. A recent patent has even claimed the usage of Rottlerin synthetic analogs, as PKC*δ* inhibitors, in the treatment of Parkinson's disease (patent application number: 20110112182).

Therefore, it cannot be stated with certainty that Rottlerin is not a PKC*δ* inhibitor; rather, the current challenge should be to discern between PKC*δ*-dependent and PKC*δ*-independent Rottlerin effects.

With the aim to explain and reconcile the conflicting findings from the literature, we propose an additional indirect mechanism by which Rottlerin can inhibit or promote certain PKC*δ* functions, depending on PKC*δ* cellular redistribution. Since PKC*δ* activation has been classically associated with transient translocation to the plasma membrane, the Rottlerin prevention of PKC*δ* membrane recruitment has been interpreted in some studies as a proof of direct Rottlerin inhibition.

However, we noticed that the Rottlerin inhibition of agonist-induced translocation of PKC*δ* has been documented in cells expressing functional caspase 3, such as neutrophils, where treatment with 10 *μ*M of Rottlerin for 15 min prior to PMA stimulation reduced the membrane localization of PKC*δ* [17], in human monocytes [18], in human promonocytic U937 cells [19], and in pancreatic acinar cells, where pretreatment for 2 h with 6 *μ*M Rottlerin inhibited by more than 60% the CCK-8-stimulated PKC*δ* translocation to particulate fractions [14].

Often, apoptosis induced by Rottlerin has been ascribed to the (apparent) PKC*δ* inhibition and interpreted as a protective role of PKC*δ* against cell death. For example, PKC*δ* was reported to act as a prosurvival factor in the MCF-7 human breast cancer cell line since inhibition of PKC*δ* by 1.5–9.0 *μ*M Rottlerin (Sigma) decreased survival in response to radiation-induced DNA damage [20]. Further, PKC*δ* has been described as antiapoptotic because its inhibition by Rottlerin promotes apoptotic death [21, 22].

However, the claimed antiapoptotic role of PKC*δ*, although cannot be excluded, has been likely confused with a PKC*δ*-independent apoptotic effect of Rottlerin; in fact, we should take into account that PKC*δ* is a caspase 3 substrate and several studies suggested that proapoptotic function of PKC*δ* is associated with its cleavage by caspase 3 in the hinge region, rendering the catalytic fragment (*δ*CF) constitutively active, thereby eliminating the membrane translocation requirement [23–25].

It is worth highlighting that, although the *δ*CF localizes to the nucleus and triggers apoptosis, its dependence on caspase 3 indicates that it cannot be the trigger of apoptosis *per se*, but it may function to amplify an apoptotic stimulus. Moreover, some reports indicate that PKC*δ* may also positively feed back on caspase 3 activation, thus inducing further apoptosis, at least in dopaminergic neurons [26].

Therefore, the hypothesized sequence of events could be as follows: Rottlerin induces apoptosis via caspase 3 activation (see below), which in turn, cleaves PKC*δ*, thus preventing PKC*δ* membrane translocation and inducing *δ*CF nuclear localization, with enforced apoptotic effect.

In this scenario, the inhibitory effect of Rottlerin on full-length PKC*δ* translocation and activity could be fully conceptually recovered (in caspase 3 expressing cells), but completely revised as far as the molecular mechanism is concerned.

Nevertheless, some exception to the rule seems to exist. For example, Zhang et al. reported that 15 *μ*M Rottlerin (Calbiochem) prohibits PKC*δ* translocation from cytosol to membrane and suppresses PKC*δ* kinase activity in caspase-3- deficient MCF-7 cells [27]. Although this finding apparently confutes our hypothesis, it is in conflict with a previous paper reporting the absence of PKC*δ* cleaved forms in MCF-7 cells after tumor necrosis factor-*α* (TNF-*α*) treatment [28].

In a further effort to reconcile divergent data, since the Zhang group did not measure caspase 3, it could be hypothesized that in their MCF-7 cell line, caspase 3 was not completely downregulated.

## 4. Rottlerin and Cancer

Rottlerin exhibits a number of therapeutic effects against a variety of cancer cells. Because of the differences in Rottlerin outcomes, cellular models, experimental strategies, and exploited pathways, the current status of the Rottlerin anticancer potential against various cancers is analyzed below under different headings.

## 5. Rottlerin and Apoptosis

Apoptosis is a particularly desirable type of cancer cell death because it causes no inflammation and can result in the dissipation of the tumor with no damage to surrounding tissues.

Apoptosis can be triggered by different stimuli and two major pathways of cell death have been identified: the intrinsic (mitochondrial or type II) and the extrinsic (receptor-mediated or type I) pathway.

The intrinsic pathway is regulated by BCL2 family members. These proteins have been characterized into three main groups that differ in how they regulate apoptosis: the antiapoptotic or survival proteins, such as BCL2 and BCLXL, the proapoptotic death signaling proteins, such as BAX and BAK, and the regulatory proteins, the so-called BH3-only proteins, such as BAD, BID, and BIM, which are essential for initiation of apoptosis [29]. The effector function of apoptotic members is mediated through release of proteins from the mitochondrial intermembrane space, such as cytochrome c, through the process of mitochondrial outer membrane permeabilization (MOMP). Cytochrome c binds apoptotic peptidase activating factor- (APAF-) 1 resulting in the activation of caspase 9, which in turn, activates caspase 3. Caspase 3 mediates cleavage of vital cellular proteins, including the nuclear enzyme poly(ADP-ribose) polymerase (PARP) [30].

The extrinsic pathway occurs independently of the BCL2 family and is triggered by ligation of death receptors belonging to the TNF family. Binding of specific ligands to the receptor induces receptor multimerization, binding of Fas-associated death domain (FADD) adapter protein, formation of the death-induced signaling complex (DISC), recruitment of the initiator caspases 8 and 10, and subsequently activation of the effector caspases 3, 6, and 7 [31].

Several early reports have shown that Rottlerin can induce apoptosis in cancer cells, such as in lung cancer, breast cancer, chronic lymphocytic leukemia, and multiple myeloma cells [20, 32–34].

However, most of these studies ascribed the Rottlerin apoptotic action to PKC*δ* inhibition; hence, as discussed previously, many of them likely should be reread in the light of the current views regarding Rottlerin's mode of action.

Obviously, the existence of PKC*δ*-unrelated Rottlerin outcomes does not reflexively imply that PKC*δ* is not involved in the control of apoptosis. In fact, although the initial results on the role of PKC*δ* in apoptotic signaling pathways were very contradictory [21, 22], recent studies support a role for PKC*δ* in apoptosis inhibition, at least in breast cancer cells. For example, it has been demonstrated that PKC*δ* downregulation by small interfering RNA (siRNA) transfection, *per se* induces apoptosis in MDA-MB-231 breast cancer cells [35].

Mechanistically, the authors found that PKC*δ* silencing led to an increase in MEK1/2 and ERK1/2 phosphorylation, indicating that PKC*δ* conceivably exerts its survival support by suppressing the Ras/MEK/ERK pathway [35].

Moreover, the existence of alternatively spliced variants of PKC*δ* with distinct functions in the apoptotic cascade has been recently discovered and could represent the switch that determines cell survival or cell death. The splice variants described so far include caspase 3 resistant full-length PKC*δ* protein and a truncated protein containing the regulatory domain, but not the catalytic one, which localizes to the plasma membrane and likely acts as an endogenous dominant negative inhibitor [36].

In recent publications, the anticancer potential of Rottlerin has been confirmed in different cancer cells and markedly different mechanisms of action have been described, again with enigmatic results as far the PKC*δ* involvement is concerned.

For instance, Zhang et al. reported that Rottlerin sensitizes MCF-7 breast cancer cells to TRIAL-mediated apoptosis by PKC*δ*-dependent inhibition of the transcription factor nuclear factor *κ*B (NF*κ*B), a major regulator of apoptosis [27].

However, we have recently reported that NF*κ*B is a direct Rottlerin target in MCF-7 cells and, although the exact mechanism of inhibition is not defined yet, it occurs in a PKC(*δ*)-independent manner [37]. Furthermore, although there is evidence that PKC*δ*, or its downstream effector PKD1, can activate NF*κ*B [38, 39], we have recently found that the PKC*δ* isoform of novel PKCs is only marginally involved in PKD1 activation in MCF-7 cells [40]. Therefore, as already noted for PKC*δ* membrane translocation, the Zhang's MCF-7 cell line strongly differs from the MCF-7 cells used in our and other studies.

Accumulating evidence suggests that Rottlerin plays a PKC*δ*-independent role in modulating apoptotic cell death signaling. For example, in colon carcinoma, 1–10 *μ*M Rottlerin (Calbiochem) sensitizes cancer cells to tumor necrosis factor-related apoptosis-inducing ligand- (TRAIL-) induced apoptosis via uncoupling of the mitochondria independent of PKC [41].

Further, PKC*δ*-independent and mitochondrial uncoupling-mediated apoptotic death has been also described in HT1080 human fibrosarcoma cells, where 0.5–10 *μ*M Rottlerin caused AIF translocation, with no involvement of caspases 3 and 9 [42].

Therefore, the mitochondrial membrane depolarization by Rottlerin cannot only affect phosphorylation and activity of proteins by reducing ATP levels, but can also account for PKC*δ*-unrelated apoptotic events.

On the other hand, it also appears that the intrinsic apoptotic pathway is not the only weapon that Rottlerin has to kill cancer cells. In fact, in a panel of human malignant tumor cells (HCT116, HT29, LNCap, PC-3, Hep3B, U2OS, SaOS2, and Caki cells), Lim et al. [43, 44] have recently reconstructed a completely different Rottlerin-triggered chain of events leading to apoptotic death. They found that 1–10 *μ*M Rottlerin (Biomol) treatment resulted in death receptor 5 (DR5) upregulation, mediated by the transcription factor CHOP/GADD153. The increase in DR5 protein, a member of the TNF receptor family, was able to trigger apoptosis even in the absence of ligand, in a PKC*δ*-, p53-, and ROS-independent manner, and, at the same time, rendered cancer cells more sensitive to the cytotoxic activity of TRAIL.

Since CHOP/GADD153 is induced by endoplasmic reticulum (ER) stress [45], the novelty of this study lies in the fact that it demonstrates that Rottlerin can induce cell death through a mechanism involving ER stress and the extrinsic apoptotic pathway, alternatively or in addition to the mitochondria targeting and the intrinsic apoptotic cascade.

Importantly for possible Rottlerin application in cancer treatment, Lim's group found that Rottlerin stimulated apoptosis preferentially in cancer cells, since no significant apoptotic effect was observed in normal fibroblasts and mesangial cells.

The observation that normal and cancer cells display different sensitivity to Rottlerin has been recently confirmed in normal pancreatic acinar cells and pancreatic adenocarcinoma MIA PaCa-2 cell line [46]. In this study the authors found that 2–10 *μ*M Rottlerin (Sigma) dose dependently induced cytochrome C release, mitochondrial membrane depolarization and stimulated not only apoptotic but also necrotic death of PaCa cells. All these events were both PKC*δ*- and Akt-independent and mediated by the Rottlerin interference in the prosurvival protein Bcl-xL, and Bim and Puma complexes formation.

In this interesting study, PaCa cells were also subcutaneously injected into nude mice. Pieces of the tumor were then transplanted into the tail of the pancreas of recipient nude mice and animals were treated daily with intraperitoneal injections of Rottlerin (0.5 mg/kg) for 4 weeks. The in vivo treatment resulted in inhibited tumor development and increased apoptosis. Importantly, there was no difference in body weight throughout the study period between control and treated groups and extratumoral tissues, for example, liver and kidney, were histologically normal.

Furthermore, Rottlerin did not activate caspase 8 in cultured pancreatic cancer cells, indicating that it stimulates only mitochondrial pathways of apoptosis. The initiator caspase 8, indeed, is a key mediator of the type I apoptotic cascade triggered by the ligand-bound Fas or TRAIL death receptors (DR4 and DR5).

Although the mechanisms underlying these different apoptotic responses remain to be determined, the findings above suggest that the Rottlerin apoptotic effect is generalized in cancer cells and can be achieved through multiple pathways, likely cell-dependent.

Moreover, the intrinsic and the extrinsic apoptotic pathways rarely overlap upstream of caspase 3 but they are not mutually exclusive and some crosstalk has been described. For example, caspase 8, the initiator of the extrinsic pathway, mediates the activation of BID, a regulatory, proapoptotic member of the BCL2 family. Once BID is cleaved by caspase 8, truncated-BID translocates from the cytosol to mitochondria, where it can mediate cytochrome c release thereby bridging type I and II apoptosis [47]. 

## 6. Rottlerin in Combination with Other Anticancer Drugs

Combination therapy refers to the use of drugs that target different signal transduction pathways, resulting in additive or synergistic effects, thereby allowing a reduction of the dose of the most toxic component and sometimes overcoming the cellular resistance mechanisms.

Rottlerin has been described to potentiate the cytotoxicity towards tumor cells of different chemotherapeutic agents.

In addition to the already mentioned Tillman's study [41], other investigators have tested Rottlerin in combination with TRAIL. Kim et al. [48] found that glioma cells, refractory to traditional radio and chemotherapy due to overexpression of inhibitors of apoptosis, such as survivin and X-chromosome-linked IAP (XIAP), are sensitized to TRAIL-induced apoptosis by subtoxic doses of Rottlerin. Yet, in this case the Rottlerin action was independent of PKC*δ*.

Rottlerin did not enhance cell death in astrocytes, further confirming that normal and cancer cells display different sensitivity to Rottlerin.

Another study reported that Rottlerin might provide therapeutic effects in malignant glioma cells when used in combination with other chemotherapeutics. For example, coadministration of 01–25 *μ*M Rottlerin (Calbiochem) with Sorafenib, an inhibitor of Raf kinase and other tyrosine kinases that target the Raf/Mek/Erk pathway, elicited an additive growth inhibitory effect and enhanced apoptosis in a panel of human malignant glioma cells [49].

Although, once again, the authors ascribed the Rottlerin effects to PKC*δ* inhibition, the combination of Rottlerin and sorafenib was confirmed to have no significant additive effect on human nonneoplastic cell lines.

Rottlerin has also been used in coadministration with imatinib, the BCR/ABL tyrosine kinase inhibitor effective for treatment of chronic myeloid leukemia. Kurosu et al. [50] found that 1 *μ*M Rottlerin (Calbiochem), independently of PKC*δ*, synergized with imatinib to induce apoptosis in leukemic cells, without affecting BCR/ABL. Since mitochondrial uncouplers, other than Rottlerin, also induced apoptosis of BCR/ABL-expressing cells in a synergistic manner with imatinib, the authors concluded that Rottlerin enhances imatinib-induced apoptosis through its mitochondrial uncoupling effect.

Although this experimentation is at its very beginning, the findings presented so far suggest that Rottlerin, as an adjuvant to chemotherapy, can enhance chemosensitivity of cancer cells and could alleviate chemotherapy-associated toxic effects.

## 7. Rottlerin and Autophagy

Autophagy is an evolutionarily conserved, multistep process that is characterized by the vesicular sequestration and degradation of long-lived cytoplasmic proteins and organelles. The resulting double-membrane vesicle, termed autophagosome, is under the control of a highly organized and hierarchical team of autophagy-related genes (ATG) proteins. The autophagosome ultimately fuses with a lysosome and the autophagic cargo is degraded by the action of acid-dependent enzymes [51].

Autophagy (microautophagy) can promote cell survival during stresses such as starvation and hypoxia, both providing a means of recycling macromolecules as an alternative energy source and eliminating toxic debris but, when the cellular stress is continuous or excessive, cell death may ensue (macroautophagy or type II programmed cell death). Then the question is perhaps quantitative: microautophagy is protective but macro-autophagy kills cells.

Because the frequent deregulation of the apoptotic pathway in cancer cells constitutes an important clinical problem in radiotherapy and chemotherapy, as an alternative route of cell death, (macro)autophagy is currently considered an important research target for new anticancer drugs.

Although Rottlerin-promoted cell death has been mainly ascribed to apoptosis, a sign of autophagy induction was firstly described about fourteen years ago in apoptosis-resistant rat and human gliomas [52]. In that study, 5–20 *μ*M Rottlerin treatment resulted in growth arrest, packaging of cellular components within membranes, and accumulation of cytoplasmic vacuoles.

Since then, other studies described Rottlerin as an inductor of autophagy, again through (apparently) different mechanisms.

In HT1080 fibrosarcoma cells, which, however, die by apoptosis, an early response to Rottlerin is the induction of autophagy by a PKC*δ*-independent mechanism, likely mediated by the mitochondrial membrane depolarization [42].

The Ozpolat and Akar's group reported that 2–4 *μ*M Rottlerin induced autophagy through inhibition of PKC*δ*/transglutaminase 2 (TG2) in a panel of pancreatic cancer cells that are known to be resistant to most chemotherapeutic agents [53, 54]. In these studies, Rottlerin- or TG2 siRNA-treated cells clearly showed formation of autophagic vesicles and a significant accumulation of endogenous microtubule-associated protein 1 light chain 3-(LC3)-II protein, a hallmark of autophagy. Since knockdown of PKC*δ* expression by a specific siRNA led to the downregulation of TG2 protein, the authors concluded that PKC*δ* regulates TG2 expression and Rottlerin induces autophagy through PKC*δ* inhibition. As a direct proof, they showed that Rottlerin caused downregulation of PKC*δ*, protein, and messenger, with no apoptosis, as judged by the lack of PARP cleavage and Annexin V positivity.

Although the role of TG2 in the control of autophagy has been also reported by other groups [55], there are some original aspects in this study that merit to be further discussed.

First, to our knowledge, there is only another study reporting that Rottlerin causes downregulation of PKC*δ*. Brown et al. [56] found reductions in the levels of PKC*δ* RNA and full-length protein both in vivo (Rottlerin-treated mice) and in cultured macrophages.

Because Rottlerin has been thought to only inhibit PKC*δ* translocation and kinase activity, these unusual findings could help explain some of the controversy that has arisen concerning the Rottlerin modulation of PKC*δ* and should be further exploited. 

The second interesting aspect of this study is the finding that Rottlerin through the PKC*δ*/TG2 axis regulates downstream targets, such as NF*κ*B and Bcl-2. In fact, among the diverse roles, TG2 is known to activate NF*κ*B [57] and hence its target gene product Bcl-2, which, in addition to protecting against apoptosis, can also prevent cells from undergoing autophagy by binding and inhibiting the autophagy-promoting protein Beclin-1.

It is of interest to recall that the inhibition of NF*κ*B by 5–20 *μ*M Rottlerin (Calbiochem) has been documented by our group in several other cell types [58, 59], MCF-7 cells included [37]. However, in MCF-7 cells, the PKC*δ*/TG2 axis cannot be operative since these cells are TG2 deficient [60], in marked contrast with the majority of pancreatic cancer cell lines, which overexpress TG2.

This therefore can be considered as an example of how Rottlerin, though targeting different signaling molecules in different cells, can converge the response on a common effect.

Interestingly, the Ozpolat and Akar's group reported that Rottlerin, through the PKC*δ*/TG2 axis also downregulated phosphorylated mammalian target of rapamycin (mTOR), a major negative regulator of autophagy [61].

The mTOR serine/threonine kinase is the catalytic component of two distinct protein complexes, mTORC1 and mTORC2. mTORC1 consists of a complex that includes mTOR and a protein known as RAPTOR (regulatory associated protein of mTOR) whereas mTORC2 consists of a complex that includes mTOR and a protein known as RICTOR (rapamycin-insensitive companion of mTOR). Only mTORC1 is allosterically inhibited by rapamycin and negatively regulated by the tuberous sclerosis complex (TSC)1/TSC2, also called Hamartin/Tuberin, tumor suppressor heterodimer.

The mTOR is an important regulator of multiple cellular functions besides autophagy, including proliferation, differentiation, tumorigenesis, and apoptosis. Increasing evidence suggests that mTOR is dysregulated in tumors [62] and agents, other than Rottlerin, which target the mTOR pathway are in various stages of clinical development [63].

However, the Ozpolat and Akar's group concluded that Rottlerin-induced autophagy is not mediated by mTOR because Rapamycin failed to induce autophagy in MDA-Panc28 cells.

Since it is now recognized that the rapamycin efficacy is limited to few cancers [64], this is probably a peculiar feature of pancreatic cancer cells.

Although the Ozpolat and Akar's conclusion that autophagy induction by Rottlerin is mTOR-independent may seem limitative in the context of this review, a recent study, performed in MCF-7 cells stably expressing enhanced green fluorescent protein- (EGFP-) LC3, has shown that Rottlerin does promote autophagy through inhibition of mTORC1 [65].

The authors found that 3 *μ*M Rottlerin (Sigma) treatment decreased phosphorylation of the mTORC1 substrates 40S ribosomal protein S6 kinase (p70S6K) and eukaryotic translation initiation factor 4E-binding protein 1 (4E-BP), only in TSC2 expressing cells, implying that mTORC1 inhibition is mediated by TSC2 activation and TSC2 upstream signaling.

Since AMPK phosphorylates and activates TSC2 to switch off mTORC1 signaling, this study opened new mechanistic perspectives in the usage of Rottlerin in cancer as an autophagy inductor, as discussed below.

## 8. Rottlerin: Looking beyond the Mitochondrial Uncoupling

There is general consensus that a major regulatory pathway of mTOR is the activation of AMPK, which can inhibit mTOR signaling by both direct and indirect mechanisms. AMPK, indeed, increases the inhibitory activity of the TSC1-TSC2 complex toward mTOR, by phosphorylating TSC2 at specific sites (Thr-1227 and Ser-1345) [66]. In addition, AMPK directly phosphorylates mTOR at Thr-2446, inhibiting its activity [67] and also directly phosphorylates the mTOR binding partner RAPTOR on Ser722 and Ser792, causing 14-3-3 binding to raptor and inhibition of mTOR(C1) [68]. 

Since AMPK has a triple inhibitory effect on mTORC1, that is, direct inhibition of mTOR and raptor and enhancement of the inhibitory action of the TSC complex, AMPK activation inevitably leads to mTOR-dependent autophagy.

AMPK is an energy sensor that promotes energy production and limits energy utilization to ensure cellular survival. In fact, AMPK is activated under conditions that deplete cellular ATP and elevate AMP levels, such as glucose deprivation, hypoxia, and mitochondrial uncouplers [69, 70].

AMP regulates AMPK by binding to the AMPK-*γ* subunit and inducing a conformational change that exposes the enzyme to upstream kinases, which activate AMPK by phosphorylation of Thr172 [71]. Moreover, AMP confers to AMPK resistance for inactivating phosphatases. At least three different upstream AMPK kinases have been recognized, the major one is the ubiquitously expressed LKB1 tumor suppressor [72].

Consistent with its uncoupling activity and reduction of cellular ATP levels, 2–10 *μ*M Rottlerin (Calbiochem) has been reported to activate AMPK in vascular cells and tissues, through a still undefined mechanism possibly involving LKB1 [73].

Unfortunately, there are not published studies on AMPK activation by Rottlerin in cancer cells.

However, although cancers are generally more dependent on glucose metabolism rather than mitochondrial respiration for energy requirements, we should consider that the AMP/AMPK axis is very sensitive and even minimal changes in the intracellular ATP/ADP ratio ensures that AMPK is activated, thanks to the activity of adenylate kinase, which catalyzes the reaction 2 ADP → ATP + AMP.

Therefore, since the Rottlerin uncoupling effects have been documented in a discrete number of cancer cells, it is tempting to speculate that AMPK activation is a widespread Rottlerin outcome.

Intriguingly, AMPK activation by Rottlerin could also account for NF*κ*B inhibition and downregulation of NF*κ*B target genes. In fact, NF*κ*B inhibition by AMPK has been demonstrated in HUVECs by both AMPK chemical activation (AICAR) and overexpression of a constitutively active AMPK [74]. Although the precise mechanism was not investigated in details by the authors, other researchers reported that AMPK enhances the activity of SIRT-1, a NAD^+^-dependent histone deacetylase, resulting in the deacetylation of several transcription factors, including NF*κ*B, which is deacetylated at Ser 310 of the p65 subunit and inactivated [75].

Importantly, this last study has been performed in nonsmall-cell lung cancer (NSCLC) cell lines.

## 9. Rottlerin and AMPK: Other Mechanistic Perspectives

As stated above, although not supported yet by experimental evidence, it is reasonable to hypothesize that the well documented uncoupling property of Rottlerin promotes AMPK activation in normal as well-as in cancer cells, regardless of the type of metabolism upon which they rely for energy production.

Among the several pathways and molecules under the control of AMPK, the AMPK/p53/p21 and the AMPK/p27 axes are particularly relevant in cancer.

The p53 transcription factor is a tumor suppressor that can be activated by cellular stress signals such as DNA damage, ultraviolet light, and oncogenes. Under normal conditions, p53 is rapidly degraded by ubiquitin-mediated proteolysis; however, posttranslational modifications such as phosphorylation stabilize p53 and enhance its DNA binding activity towards promoter regions of target genes.

It has been demonstrated that both chemical activation of AMPK [76] and expression of constitutively active AMPK [77] result in phosphorylation of human p53 at Ser15, p53 stabilization, and enhanced transcriptional activity.

AMPK and p53 are linked by a mutual activation loop, by which AMPK stabilizes p53, which, in turn, activates AMPK through complex and incompletely defined mechanisms, which, however, clearly lead to inhibition of mTOR and induction of autophagy [78, 79].

As far as the AMPK/p27 axis is concerned, recent studies reported that activated AMPK phosphorylates the cell cycle inhibitor p27 at Thr 198, thus stabilizing p27 and prolonging its activity [80].

AMPK can also induce the transcription of the p27 gene by a mechanism involving in sequence AMPK/SIRT-1/FOXO pathway. As mentioned above, AMPK enhances the activity of SIRT-1, resulting in the deacetylation of several transcription factors, Forkhead box class O (FOXO) included. However, FOXO deacetylation by SIRT-1 enhances its transcriptional activity, resulting in increased expression of p27 [81] and induction of autophagic flux [82].

Notably, in addition to inhibiting the cell cycle progression and to protecting against apoptosis, p27 has also been implicated in the induction of autophagic death [83].

## 10. Antimetastatic Effects of Rottlerin

Migration and adhesion of tumor cells are the most important prerequisites for the formation of metastasis. Metastatic process involves the separation of cancer cells from the primary tumor, migration into the extracellular matrix, blood vessel invasion, adhesion to endothelium, and extravasation and growth in a secondary organ.

Cell adhesion, the binding of a cell to extracellular matrix or another cell, involves cell adhesion molecules such as cadherins and integrins.

Cadherins are Ca^2+^-dependent type-1 transmembrane proteins that participate in the maintenance of proper cell-cell contacts. E-cadherin is the major adhesion molecule of epithelial cells. E-cadherin is considered a tumor suppressor protein, preventing cancer cells from detaching from a primary tumor mass, traveling through the bloodstream, and invading other tissues [84]. 

Integrins are a large family of heterodimeric transmembrane glycoproteins, composed of noncovalently linked *α* and *β* subunits that mediate mainly cell-matrix interactions. In addition, integrins have been considered as signaling receptors essential for proper cell growth and motility [85]. Alterations in integrins composition have been correlated with malignant progression and tumor invasion in vivo and in vitro [86].

Cell migration in metastasis involves turnover of focal adhesion complexes, which link the intracellular cytoskeleton and the transmembrane integrins, which, in turn, anchor cells to the extracellular matrix and relay signals therein.

Focal adhesion complex consists of several component proteins, including focal adhesion kinase (FAK), vinculin, and paxillin. Among these, FAK and its activated form, phosphoFAK (Tyr397), plays crucial roles in mediating the assembly and disassembly of the complex. Once activated by clustering of integrins, pFAK activates downstream GTPases RhoA and Rac-1, leading to polymerization of actin and formation of stress fibers [87].

Rottlerin has been demonstrated to affect cell motility and adhesion by modulating the expression of a number of cellular adhesion molecules involved in the metastasis process. 

Again, most studies ascribe the Rottlerin effects to direct inhibition of PKC*δ*.

The only study reporting a PKC*δ*-independent antimetastatic potential of 10 *μ*M Rottlerin (Biomol) has been recently published by Lin et al. [88], who demonstrated that Rottlerin reduced cell motility and cell adhesion to substratum matrix of follicular thyroid carcinoma cells. Moreover, Rottlerin treatment decreased protein levels of integrin *β*1, FAK, and paxillin with consequent disassembly of the focal adhesions. Rottlerin also reduced expression level and activity of Rac-1 and Rho GTPases, which was accompanied by disrupted actin stress fiber formation.

Very similar results had been previously obtained in renal carcinoma, prostate cancer, and colon carcinoma cells, although ascribed to PKC*δ* inhibition [89–91].

Cell motility is just a component of the invasion process. Invasion of the surrounding tissue by tumor cells also involves degradation and remodeling of extracellular matrix. These processes are mediated by two types of proteolytic enzymes: the plasminogen activator system components and the matrix metalloproteinases (MMPs).

In recent works, again through PKC*δ* inhibition, 2.5–10 *μ*M Rottlerin treatment decreased MMP-12 levels and invasiveness of tenascin-stimulated glioma cells [92] and caused a marked decrease in PMA-induced MMP-9 secretion and invasiveness in MCF-7 human breast cancer cells [93].

Similarly, 5 *μ*M Rottlerin (Calbiochem) had been previously found to markedly inhibit both FGF2- and TPA-induced MMP-9 secretion in MCF-7 cells as well as basal and PMA-induced MMP-9 expression in pituitary adenoma cells [94, 95].

Although the mechanisms underlying the Rottlerin anti-invasion properties are relatively poorly understood at present, as many prometastatic genes are regulated by NF*κ*B (MMP-9 and E-Cadherins, among others), it is reasonable to propose that the suppressive effect of Rottlerin on NF*κ*B activation, rather than (or in addition to ?) PKC*δ* inhibition, is determinant in the blockage of metastatic cell functions.

## 11. Conclusion

This review, focused on the work performed so far on Rottlerin anticancer properties, demonstrates that Rottlerin does not function by virtue of interacting with a single well-defined molecular target, but supports the concept that it acts upon numerous biochemical and molecular mechanisms.

There is also the remote possibility that the pleiotropic Rottlerin effects could be due to impurities present in the commercial preparations. However, as specified throughout this review, the different effects described have been observed by using commercial Rottlerin from different companies, at doses ranging from 1 to 25 *μ*M. Moreover, we verified that the commercial preparation from Calbiochem is 100% pure, as evidenced by the single peak in the capillary zone electrophoresis electropherogram (unpublished data).

Another complicating factor is the debated activity of Rottlerin towards PKC*δ*, an old question that is still far from being clarified.

Therefore, it is extremely difficult, if not impossible, to formulate an unifying hypothesis. This is due, not only to the different mechanisms of action described as well as to the cell specificity, but also to the existence of possible crosstalks and common effectors among the different cell death pathways. While there is still much to learn about the molecular mechanisms underlying the multiple Rottlerin anticancer effects, it is also true that the ability to attack multiple targets are in favor of Rottlerin being developed as a drug for prevention and therapy of various cancers. In the last years indeed, the tendency to adopt a multitarget-based anticancer therapy is becoming imperative to decrease the probability that cancer cells will develop chemoresistance.

Although the Rottlerin potency in vivo remains almost unknown, its prospects are greatly anticipated. It might therefore be expected that the Rottlerin diversified anticancer effects that have been already shown in a variety of tumor cell types, will soon provide strong preclinical data for the justification of clinical studies in humans as a metastatic cancer preventive/curing approach.
